# Effect of Cardinal Directions on Gall Morphology and Parasitization of the Gall Wasp, *Cynips quercusfolii*

**DOI:** 10.1673/031.011.16901

**Published:** 2011-12-12

**Authors:** Mohammed Reza Zargaran, Mohammed Hassan Safaralizadeh, Ali Asghar Pourmirza, Orouj Valizadegan

**Affiliations:** Urmia University, Faculty of Agriculture, Department of Entomology, Uremia University, West Azerbaijan, Iran

**Keywords:** oak, parasitoids

## Abstract

This survey investigated the relationship between gall morphology and some fitness components in the asexual generation of *Cynips quercusfolii* L. (Hymenoptera: Cynipidae). Results showed that larger *C*. *quercusfolii* galls were formed on the south side of oak trees *Quercus infectoria* Olivier (Fagales: Fagaceae). Larval chamber diameter in the gall was similar, but gall diameter and gall wall thickness varied with the location of the gall on the tree. *Cynips quercusfolii* was attacked by parasitoids, and the south-facing galls suffered significantly lower parasitoid attacks. Thickness of gall walls and parasitism rate were negatively correlated. Mean gall diameter and gall wall thickness were significantly larger in south-facing galls than other directions, but the difference in the mean larval chamber diameter was not significant. These results suggest that the position of galls on the tree affected gall wall thickness, and this plays an important role in parasitoid attacks. These results suggest that *C*. *quercusfolii* prefer to attack the south side of oak trees, and selection of this side by wasps led to formation of larger galls with thick walls that decreased parasite attack, which will affect growth and survival of wasp larvae.

## Introduction

Iran lies at the eastern limit of the Western Palaearctic ecoregion, and recent surveys confirm that this country has a rich Cynipid fauna (Azizkhani 2006; Sadeghi et al. 2010) and includes many of the widespread Western Palaearctic species, including *Cynips quercusfolii* L. (Hymenoptera: Cynipidae) (Melika et al. 2004).

The asexual generation of the cynipid wasp *Cynips quercusfolii* causes hemispherical leaf vein galls on oak trees, *Quercus infectoria* Olivier (Fagales: Fagaceae). Galls of *C*. *quercusfolii* are distributed in oak forests of West Azerbaijan in Iran. The asexual generation of *C*. *quercusfolii* forms galls on oak with a hemispherical single larval chamber on the upper leaf surface. The *C*. *quercusfolii* oak gall has a single chamber and a single attacking parasitoid. In Sardasht, the galls appear successively from mid—August to September, and each gall matures within one month. Larvae grow rapidly in late September to early October, pupate in late September to mid—October, and adults emerge from the galls in late October. Some mature galls fall from the leaves, while many immature galls that are attacked by parasitoids remain on the leaves (Zargaran et al. 2008).

Typically the sexual generation occurs in the spring and the asexual female generation is in the fall. Structurally, galls induced by sexual or asexual generation of gall wasps are divided into two larval chambers surrounded by an outer layer. Larval chambers are alike in most galls. Surrounding each larval chamber is a mass of nutritive cells surrounded with a single layer of parenchyma cells. In most galls, these two layers are covered with a third layer of sclerenchyma cells (Stone and Cook 1998). Normally, nutritive cells provide the food required for gall wasp larva. However, parenchyma cells of the gall may be converted to nutritive cells when needed, compensating food supply shortages (Stone et al. 2002). The outer part of a gall is comprised of the central part of the gall to the sclerenchyma layer and beyond it. The outer parts of galls are used as characters to specify various gall wasps (Schonrogge et al. 2000). The outer structures of the galls include layers of woody or spongy (sometimes with hollow spaces) tissue, superficially covered with thick layers of resins or hairs, lint, or spines (Price et al. 1987; Stone and Cook 1998). The size of a gall is most likely directly related to many factors such as wasp potential fecundity, which is positively correlated with gall size (Kato and Hijii 1993; Gilbert et al. 1994).

Parasitoids and probably inquiline attacks are principal factors in the mortality of the cynipids (Washburn and Cornell 1981). Gall size, size of larval chamber, and wall thickness affect the ability of parasitoids to successfully attack the cynipids (Washburn and Cornell 1981; Schonrogge et al. 1996). These factors affect parasitoid attack on galling insects, and are related to the morphological traits of the gall.

Evaluation of the relationship between gall morphology in different geographical directions and climate, such as avoidance of parasitoid attack, will provide a better understanding of the population dynamics of *C*. *quercusfolii* galls and probably for other cynipids. The objective of this study was to evaluate the relationships between climate and *C*. *quercusfolii* gall placement within *Q*. *infectoria* canopies in relation to gall morphology and parasitoid activity.

## Materials and Methods

Sampling was carried out in the oak forest of West Azerbaijan province in the Sardasht and Piranshahr City in two locations (with similar plant covering): Ghabre-hosein (36^°^ 28′ N, 45^°^ 18′ W) with a humid—cold climate, and Rabat (36^°^ 14′ N, 45^°^ 33′ W) with a Mediterranean—humid climate, based on Dumarten's climate classification method. Table 1 shows the environmental conditions of these two locations from July to December 2010. The oak forest of West Azerbaijan is composed mainly of *Q*. *infectoria* (comprising 90% of plant cover); this species is predominant.

The optimal number of samples was determined according to the formula of Southwood and Henderson (2000):


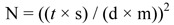


where *t* is student's *t*—test using standard statistical tables, d is the predetermined confidence limit for the estimation of the mean express as a decimal, m is sampling mean, and s is the standard deviation.

The best number of samples was determined to be 20 trees. On each tree, as a unit of sampling, galls were collected on one branch (branch length was 90 cm) in each cardinal direction (north, south, east, west)—totaling four branches in one tree—on 20 October 2010. The effect of cardinal directions on *C*. *quercusfolii* gall abundance was analyzed with these data. Additionally, a total of 400 *C*.*quercusfolii* oak galls of (100 galls in each direction) were surveyed from 20 *Q*. *infectoria* trees (trees with 3 m in height and open canopy) in each location on 20 October 2010 by pruning branches with galls within 4m of ground level, for a total of 800 galls in the two locations. The galls were placed in nylon bags along with the branches pruned from each direction, and were kept under outdoor conditions. Adult cynipids and parasitoids emerging from the galls were collected every day for 10 days and were stored in 75% ethyl alcohol. Only parasitoids that reached the adult stage were surveyed and identified to the species level.

**Table 1.**
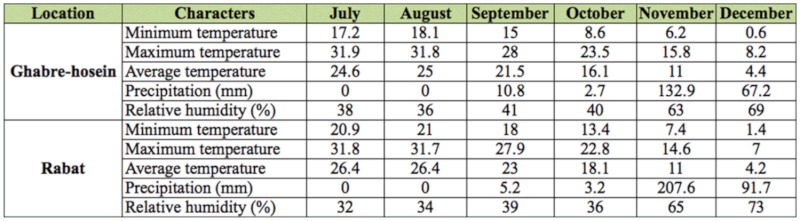
Descriptions of collecting sites in west Azerbaijan, 2010.

Ten days later, the morphological traits of these collected galls were studied. All galls formed on a leaf located in a middle position on each shoot were sampled, because gall morphologies might be affected by variation in leaf quality.

The galls were then dissected, and the percentage of galls parasitized was calculated based on this formula: [number of extracted parasitoids in each side/(100 — the empty galls)]* 100.

Mortality of *C*. *quercusfolii* in this study was only caused by parasitoids. After calculating the rate of parasitism, the gall size, gall wall thickness, and larval chamber diameter were measured as follows: galls were cut open longitudinally and examined under a dissecting microscope, the largest and smallest diameters of each gall were measured with digital calipers to the nearest 0.02 mm,and the diameter of the larval chamber was measured with an ocular micrometer to the nearest 0.08 mm. Then, gall wall thickness was defined as such: gall wall thickness = (gall diameter — larval chamber diameter)/2. Empty galls and galls with a hole at the time of dissection were also recorded.

**Table 2.**
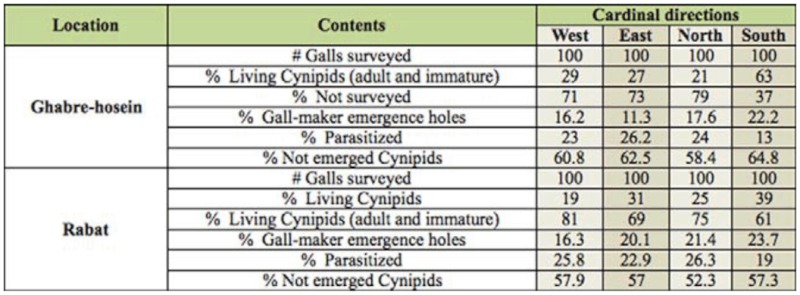
Contents of galls and the percent parasitized *Cynips quercusfolii* galls in two locations, 2010.

**Table 3.**
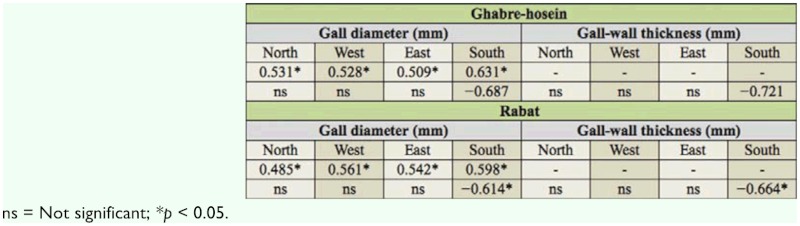
Relationships of gall diameter vs. gall wall thickness and percentage parasitism.

## Data analyses

The galls with living inhabitants were used for the analysis of gall morphology. Pearson's correlation coefficient was used to test the relationship between paired trait variables in the four cardinal directions: gall diameter vs. gall wall thickness, gall diameter vs. larval chamber diameter, and gall wall thickness vs. parasitism. To examine the changes in gall morphology, the mean values for gall diameter, gall wall thickness, larval chamber diameter, percent of parasitism, and geographical direction of gall occurrence were tested among sampling places using a one—way ANOVA.

The data were tested for normality by Shapiro-Wilk (data less than 50); if significant value was less than α= 0.05 then the data were considered not normal. Thus, data were transformed by (√x + 0.5).

Student's *t*—*test* was used between gall diameter vs. gall wall thickness vs. larval—chamber diameter and percentage parasitized in the locations. When necessary, Tukey's Honestly Significant Difference test was used for multiple comparisons of mean values. If homogeneity of variances between groups was not certain, the Kruskal-Wallis test was used. The emerging rate of *C*. *quercusfolii* from each direction was calculated for the determination the stability in each direction.

## Results

**Figure 1.**
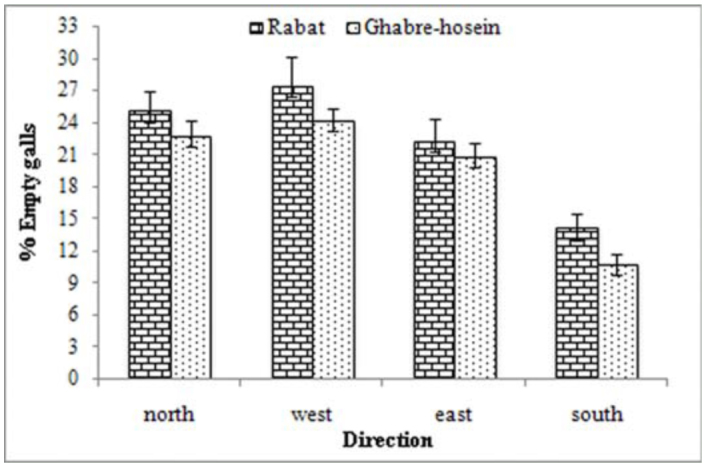
Percent of empty galls at different directions in Rabat and Ghabre-hosein station. High quality figures are available online.

There was a significant difference in the mean number of collected galls from all directions in both humid—cold (df = 3; *p* < 0.05) and Mediterranean—humid climate (df = 3; *p* < 0.05). The mean number of galls was higher in the southern direction (in humid—cold: mean ± SD; 4.85 ± 0.72, and in Mediterranean—humid: mean ± SD; 4.12 ±0.34) than the other directions in each two stations; the northern (in humid—cold: mean ± SD; 3.16 ± 0.72, and in Mediterranean—humid: mean ± SD; 3.09 ± 0.51), eastern (in humid—cold: mean ± SD; 3.27 ± 0.89, and in Mediterranean—humid: mean ± SD; 3.18 ± 0.57), and western (in humid—cold: mean ± SD; 3.11 ± 0.41, and in Mediterranean—humid: mean ± SD; 3.19 ± 0.61) directions were in the same statistical group (Tukey's test; *p* < 0.05 in each two stations). In general it seems that oak gall wasp females prefer to lay eggs on the southern side where exposure to the sun is most intense. The lowest number of empty galls (Figure 1) and also the highest of gall—maker emergence holes (Table 2) were recorded in south direction in Mediterranean—humid and humid—cold climate, and was significantly different from the other directions.

**Figure 2.**
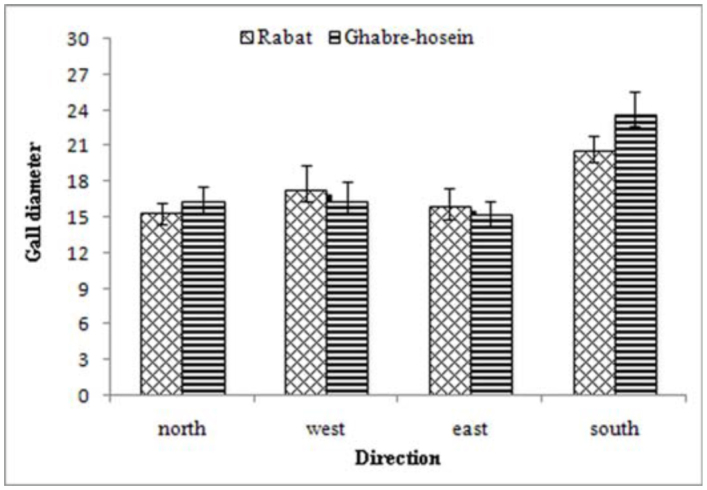
Mean gall diameter of galls at different directions in Rabat and Ghabre-hosein station. High quality figures are available online.

Mean gall diameter and also gall wall thickness among different cardinal directions differed from each other and were significantly different (df = 3; *p* < 0.05) in each two stations. The highest mean values of the gall diameter were recorded in the south direction both in humid—cold and Mediterranean—humid climates (Figure 2). Also, the highest mean values of gall wall thickness were seen in humid±cold and Mediterranean—humid climate in the galls occurring in the south direction, and the least of above listed morphological traits was seen in other directions. North, west, and east directions were placed in one statistical group (Tukey's test; *p* < 0.05 in each two stations) that were similar in gall diameter and gall wall thickness value. But the larval—chamber diameter of the collected galls in all directions was not significant among them in the two stations (df = 3; *p* > 0.05). These results suggest that selection of the cardinal directions by *C*. *quercusfolii* affect the morphological traits gall diameter and gall wall thickness, but does not affect larval—chamber diameter.

In this survey, three solitary parasitoid species were recorded from asexual galls of *C*. *quercusfolii*: *Sycophila biguttata* Swederus (Hymenoptera: Eurytomidae) from humid—cold and Mediterranean—humid climate, *Eurytoma* brunniventris Ratzeburg(Eurytomidae) from humid—cold climate, and *Megastigmus dorsalis* Fabricius (Torymidae) from humid—cold and Mediterranean—humid climate. The parasitoid species *E*. *brunniventris* was not seen at Mediterranean— humid climate.

Of these three parasitoids *E*. *brunniventris* was the most abundant. The mean percentage of parasitism was significantly lower (df = 3, *p* < 0.05) (Table 2) on south-facing galls than in other directions, but showed no significant difference among the east, west, and north direction (placed in one statistical group). The value of Pearson's correlation coefficient between gall diameter and gall wall thickness showed a significant positive correlation *(p* < 0.05) for all galls in the two stations. Also, gall diameter and larval chamber diameter were not significantly correlated *(p* > 0.05). The mean percentage of parasitism with gall diameter and also with gall wall thickness showed significant negative correlation *(p* < 0.05) only for the galls on the south side of the trees in humid—cold and Mediterranean— humid climate (Table 3) and was not significant between other directions *(p* > 0.05). These results suggest that *C*. *quercusfolii* prefer to select the south side of trees for gall forming, because galls in this direction are large in comparison with other galls and presence of a thick gall wall decreases the parasitism rate. Thus, it might increase the survival of the cynipid wasps, especially the larvae.

Effects of differences in climate on gall morphology and parasitism were examined in the two humid—cold and Mediterranean— humid climates. Mean gall diameter was larger in the galls that were formed on south side of trees in humid—cold than those in Mediterranean—humid climate (Student's *t*— test; *t* =2.58, p < 0.05), but comparison between the other directions (north, east, and west) using Student's *t*—test were not significant *(p* > 0.05). The larger galls provide more food and protection for the parasitoid larvae. However, mean larval chamber diameter was not significantly different between directions. Gall wall thickness between the galls on the south side of trees in two humid—cold and Mediterranean—humid climates was significant (Student's *t*—test; *t* = 0.59, *p* < 0.05), but this morphological trait did not differ in other directions. Also, the percentage of parasitism in the south direction in humid—cold and Mediterranean—humid climate was significant (Student's *t*—test; *t* = 3.56, *p* < 0.05) and this was higher in Mediterranean—humid, while the number of species of parasitoids was greater in humid— cold. The structure of the galls is an important factor affecting parasitism. Also, the larger galls that were formed in humid—cold climate have thicker walls, which may be one of the reasons for the lower the percentage of parasitism.

## Discussion

These results show that larger galls with thicker walls were formed on the south side of the oak trees in humid—cold climate. In addition to genetic factors, other factors such as location of gall formation and climate can affect gall size. It is likely that larger galls can be effective in survival of the cynipid wasp larvae in at least three ways: (1) greater food availability, (2) maintenance of favorable environmental conditions within the gall, and (3) increased protection against natural enemies including parasitoids. Gall size affects the morphological traits of some galling insects (Weis and Abrahamson 1985; Kato and Hijii 1993; Gilbert et al. 1994), and larger gall size may directly affect the percentage of parasitism.

The higher numbers of holes in the galls on the south side of the oak trees show that these gall wasps successfully exited and wasp development was completed. The percentage of parasitoid attacks was lower for the galls established on the south side of the trees than on three other directions; gall wall thickness is the main reason for this result.

One of the rare parasitoids that attacks smaller host larvae, *Eurytoma brunniventris*, was not found in Mediterranean—humid climate. Many factors other than the presence of a host limit the distribution of parasitoids and herbivores, but the most obvious one is climate (Barratt 2004). Weis et al. (1985) reported that *E*. *gigantea* spends a great amount of time probing galls too big to penetrate, which in turn leads to a decrease in foraging efficiency when many large galls are present. Moreover, the chance of successfully penetrating into a gall is affected by the thickness of the gall wall and the length of the parasitoid's ovipositor. However, larger galls represent a more profitable resource for those natural enemies that can gain access to them (Williams and Cronin 2004).

Cynipid wasps have been shown to control the physiology of gall tissues (Abe 1997; Schonrogge et al. 2000; Ronquist and Liljeblad 2001). Clearly, a food supply of high nutritional quality is required for the growth and development of the wasp larvae; therefore, gall formation on the south side of trees will enhance survival of the larvae. In our results, the lower number of oak galls on east, north, and west sides of trees may be due to the reduced oviposition of oak gall wasps or inappropriate conditions for continued larval growth. Although the egg is often positioned on the plant surface with only the tip inserted into the epidermis, sometimes the whole egg is embedded in plant tissue. The attacked plant tissue eventually produces a gall, inside of which the wasp larva feeds on a specially developed tissue (Vardal et al. 2003). This may be because of warmer conditions of the southern side compared with others, which suggests that the preference of wasps for warmer places for oviposition has a selective advantage because development of a gall is more likely to occur.

Parasitoids depend on a series of adaptations to the ecology and physiology of their hosts and host plants for survival, and are thus likely to be highly susceptible to changes in environmental conditions (Hance et al. 2007). In many cases, the galls have been found to contain higher concentrations of defense chemicals than normal plant tissues. Some of them are phenolics that have a defensive role, while others are tannins that have been shown to act as feeding deterrents, growth inhibitors, and toxins against insect herbivores (Nyman and Julkunen-Tiitto 2000). It is possible that secondary plant compounds accumulate in the south side of trees.

Large galls also have characteristics that deter parasitoid attacks (Weis and Abrahamson 1985; Price and Clancy 1986). If small *C*. *quercusfolii* individuals are large enough for parasitoids, the higher rate of parasitoid attack on smaller galls in three directions (east, west, and north) suggests that parasitoids can oviposit more easily on the host in smaller galls. Gall size was correlated both with gall wall thickness and parasitism rates. A thick gall wall can protect the wasp larvae against parasitoid attack, as the ovipositor cannot penetrate the galls.

Life—history traits affected by cold exposure and extreme temperatures can reduce endosymbiont populations inside parasitoids, and eventually eliminate populations of endosymbionts that are susceptible to high temperatures (Hance et al. 2007). One good measure of gall fitness is the probability that a larva will survive. In some cases this is greatly influenced by the size of the gall that gall wasps induce in the plant, which depends on several different factors. In places where the parasitoids are abundant there is strong selection for large galls; larvae are protected because large galls have walls that are too thick for wasp ovipositors to penetrate. Indirect effects of climate conditions affect gall size and increase the ability of the gall wasps to produce large galls. Although gall wasps do have some genetically—determined influence over gall size, the plant itself plays a greater role in determining gall size (Weis and Abrahamson 1998).

Climate change can influence plant productivity both directly and indirectly. Indirectly, climate change can affect the phenology of the relationship between climatic settings and periodic biological events of certain regions (Thomson et al. 2009). Romeis et al. (2005) reported that parasitism levels by *Trichogramma* varied greatly among different habitats, plants, or plant structures. Considering the low parasitism rate in humid—cold climate, we hypothesize that a habitat's annual climate patterns might determine oviposition rates, lifetime fecundity, and the number of generations in a year. Abiotic factors such as soil quality, weather, humidity, and others affect the structure the galls (Price 2005), and their direct or indirect effects must not be ignored.
